# Supplementary figures and images for: Zbtb14 regulates monocyte and macrophage development through inhibiting pu.1 expression in zebrafish
Source: eLife. 2022 Oct 7;11:e80760. doi: 10.7554/eLife.80760 (PMC9566859; doi:10.7554/eLife.80760)

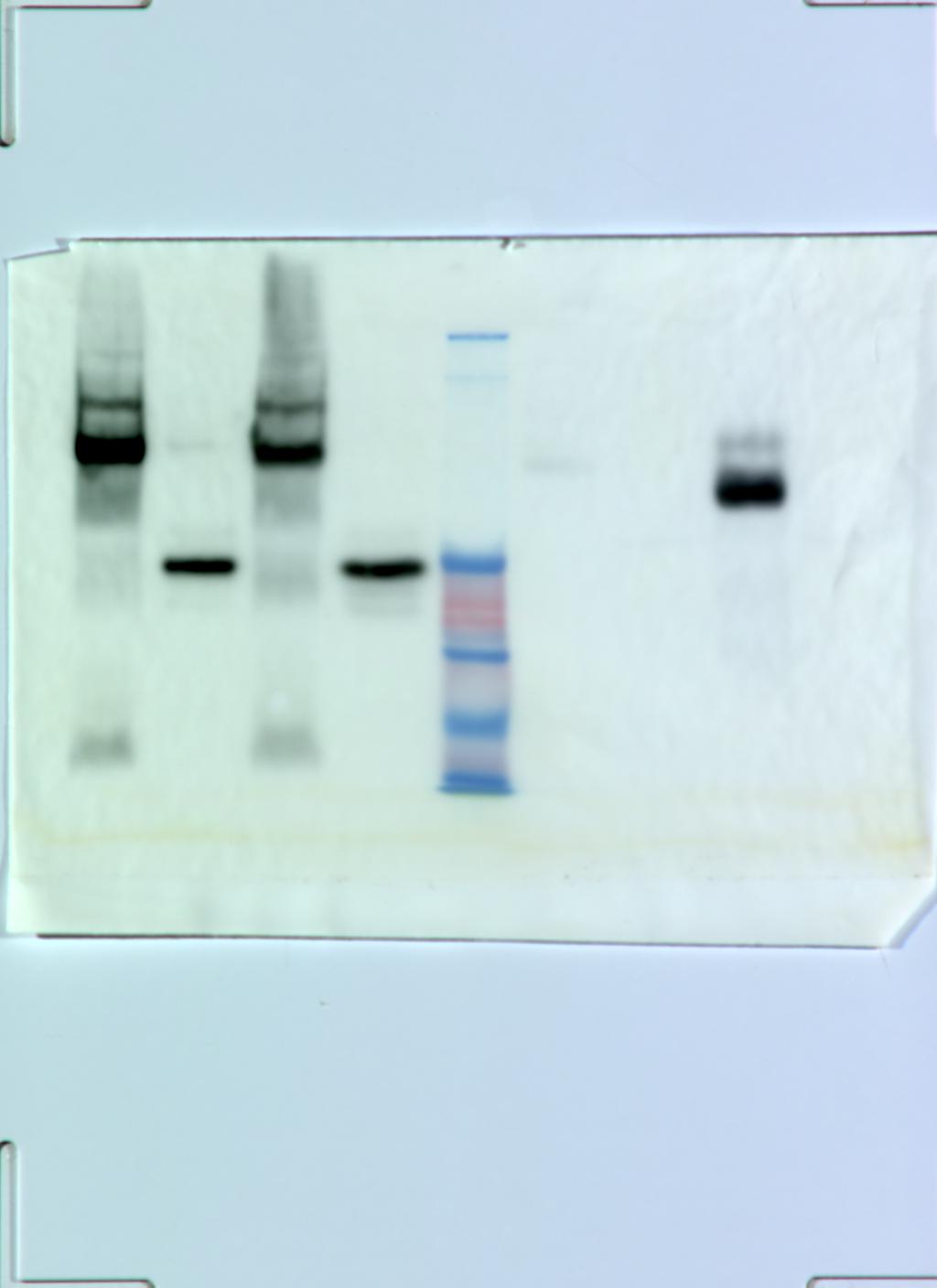

Supplement: Figure 1—source data 1. [file elife-80760-fig1-data1.zip › Figure 1-source data 1/Fig1D-HA-mutant.jpg]

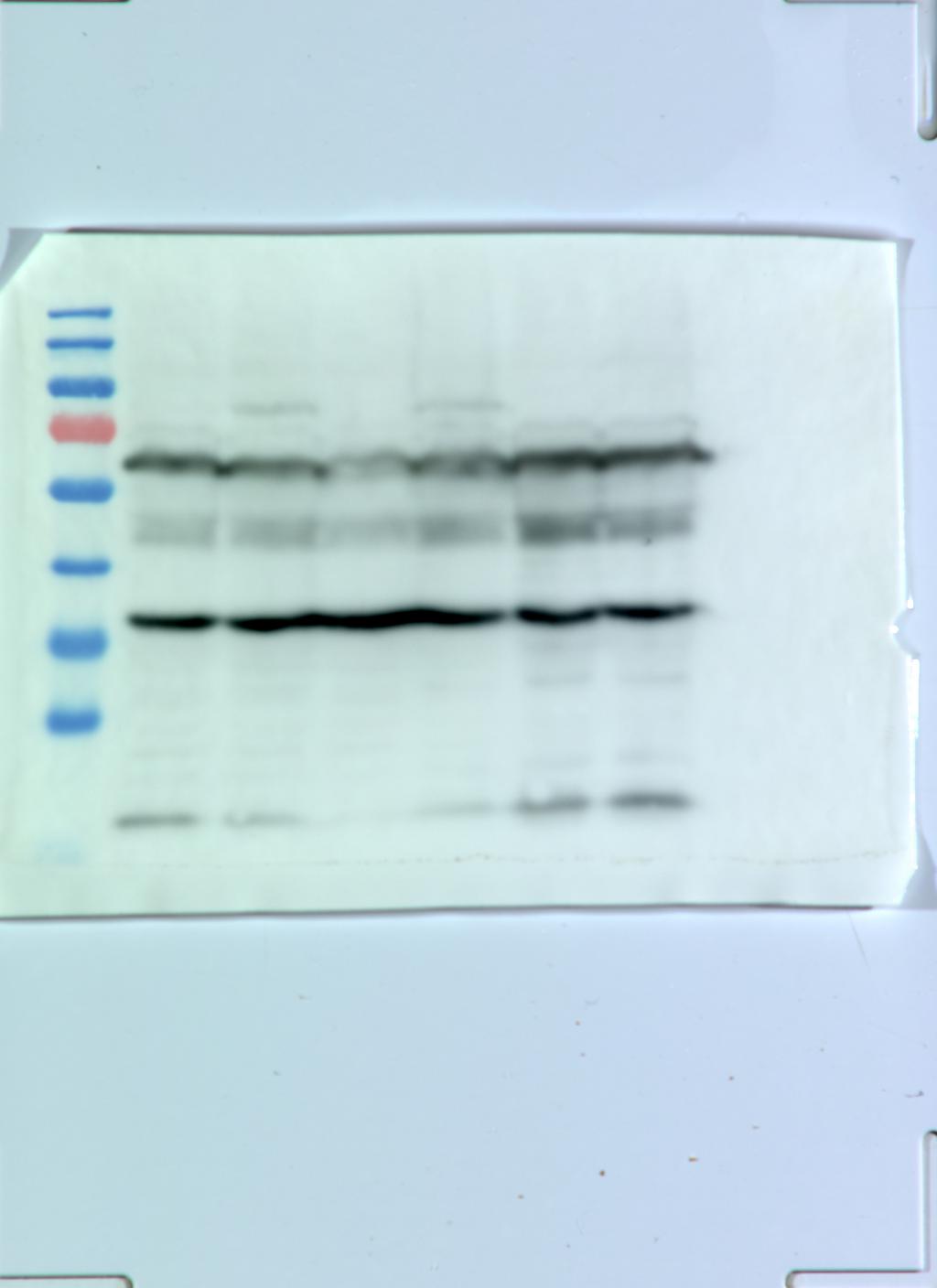

Supplement: Figure 5—source data 1. [file elife-80760-fig5-data1.zip › Figure 5-source data 1/Fig5A-GAPDH.jpg]

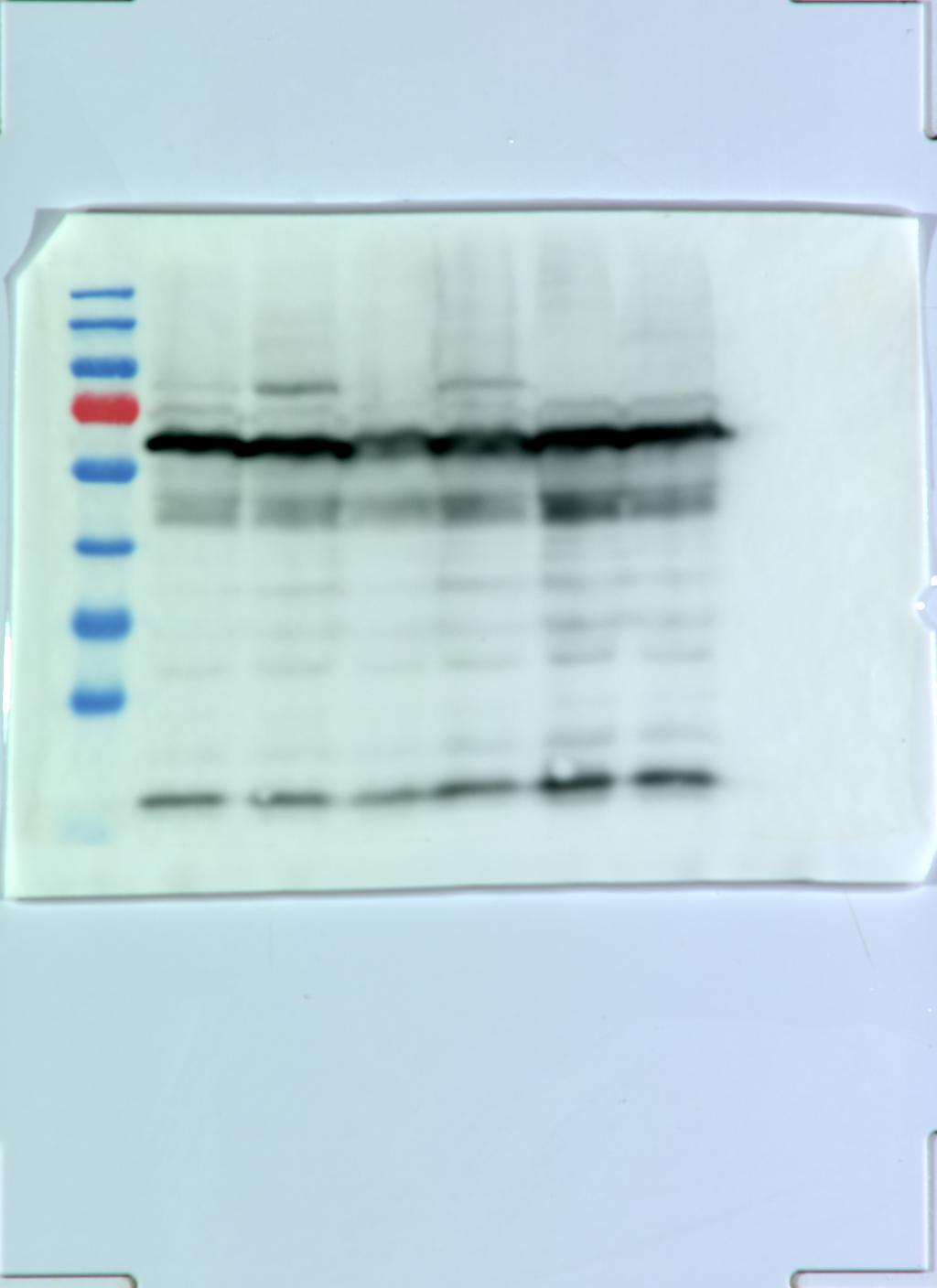

Supplement: Figure 5—source data 1. [file elife-80760-fig5-data1.zip › Figure 5-source data 1/Fig5A-HA-zbtb14 mu.jpg]

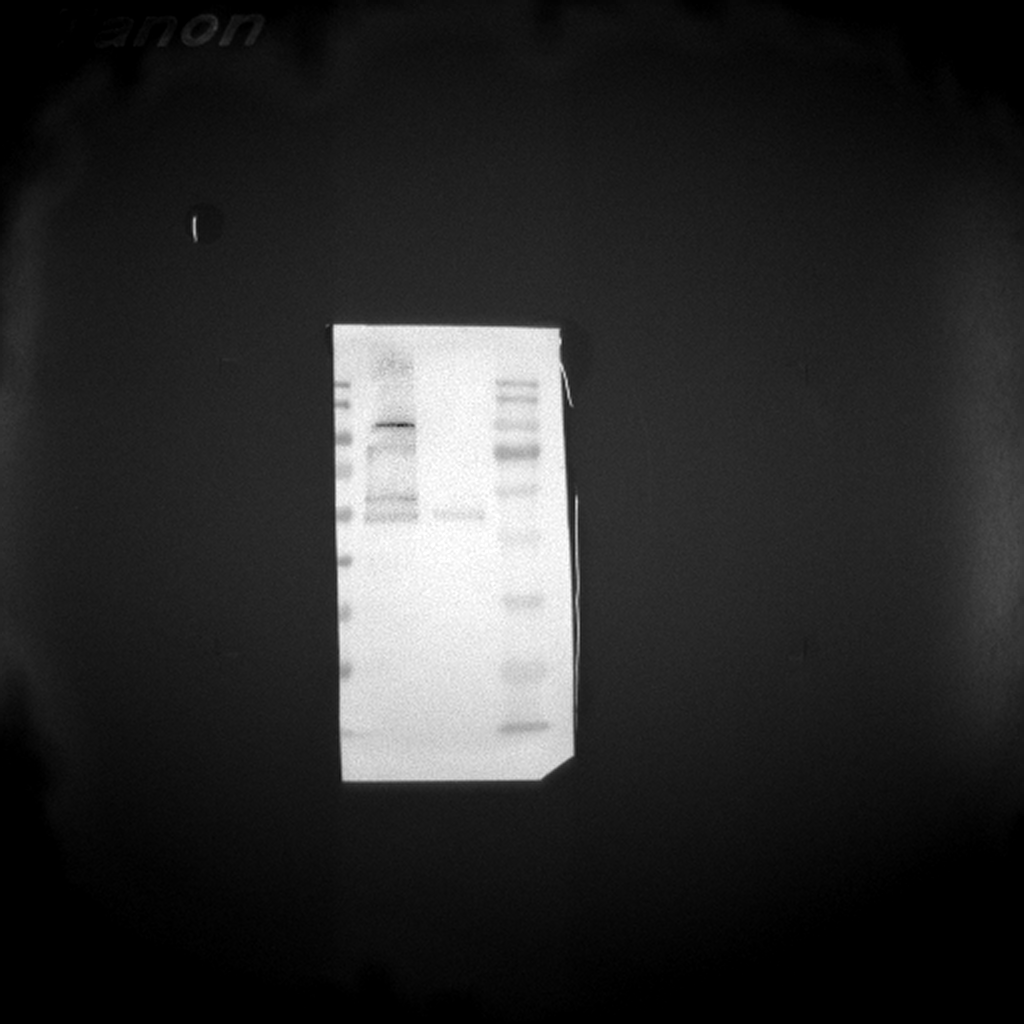

Supplement: Figure 5—source data 2. [file elife-80760-fig5-data2.zip › Figure 5-source data 2/Fig5B-GFP-IP.tif]

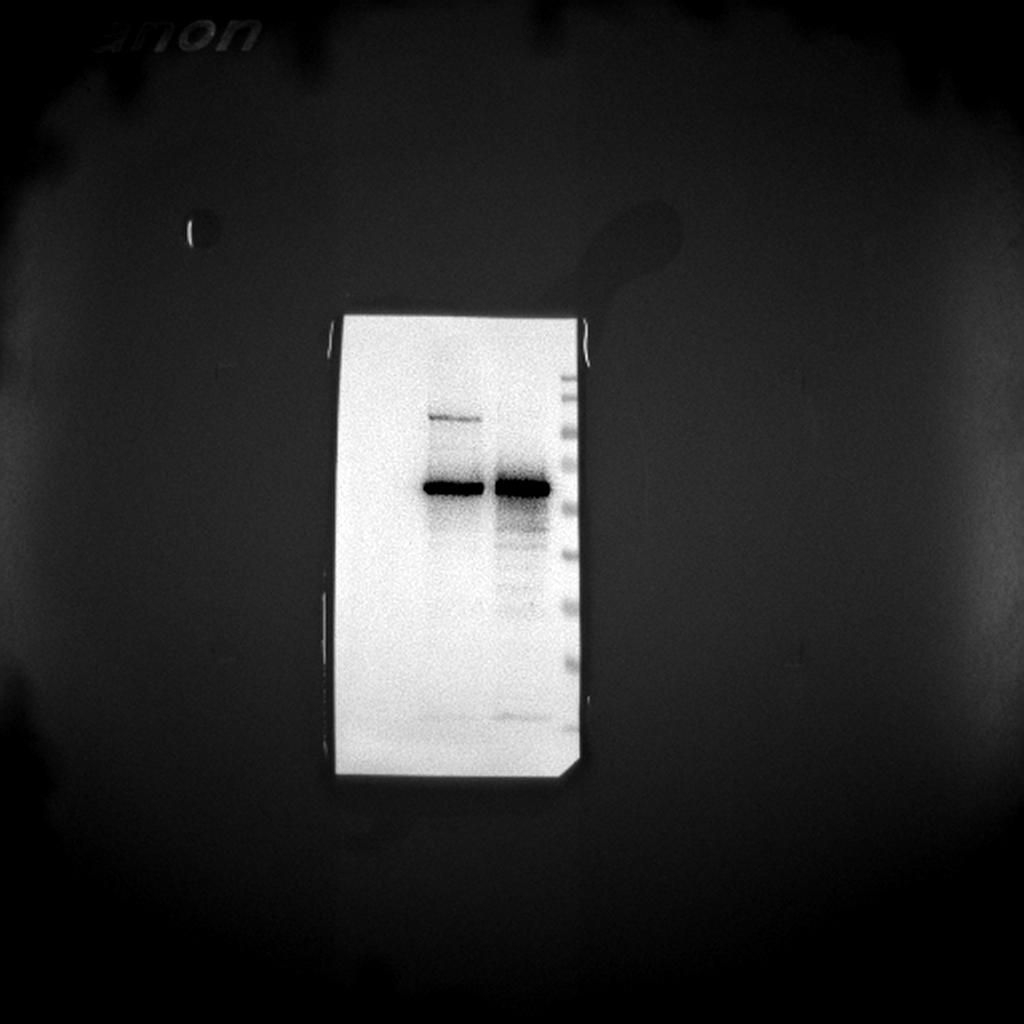

Supplement: Figure 5—source data 2. [file elife-80760-fig5-data2.zip › Figure 5-source data 2/Fig5B-HA-INPUT.tif]

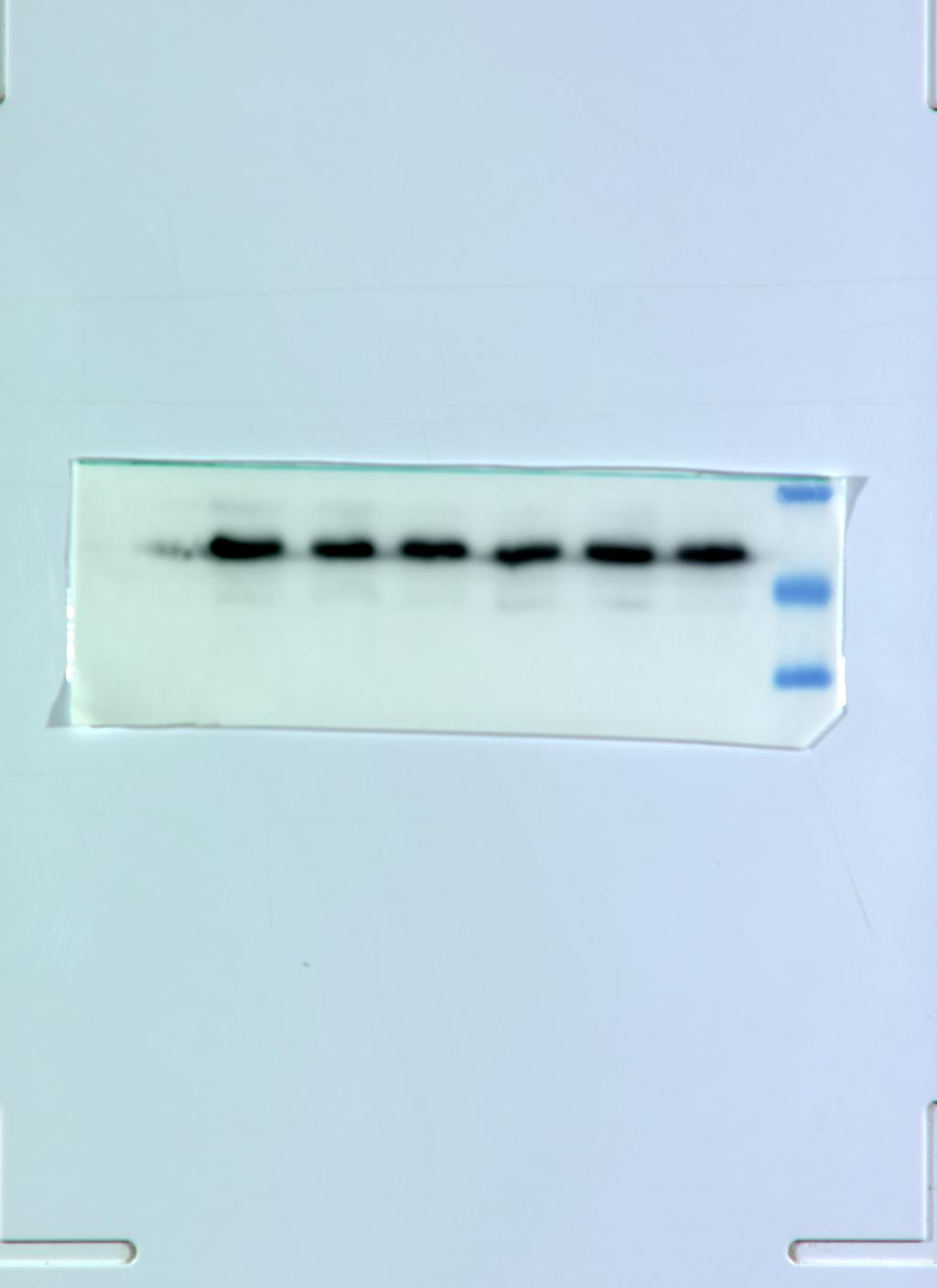

Supplement: Figure 6—source data 1. [file elife-80760-fig6-data1.zip › Figure 6-source data 1/Fig6D-left-GAPDH-1.jpg]

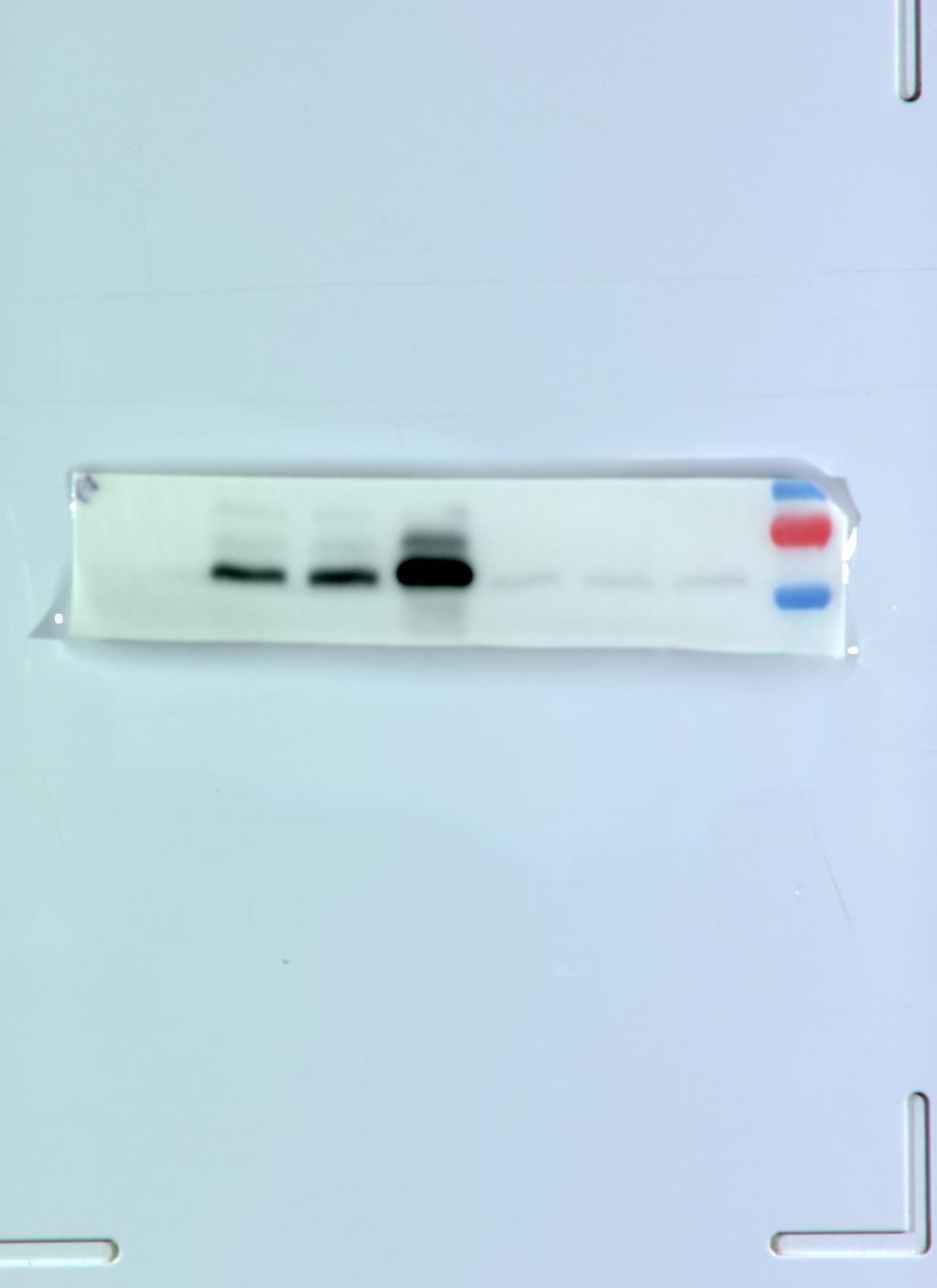

Supplement: Figure 6—source data 1. [file elife-80760-fig6-data1.zip › Figure 6-source data 1/Fig6D-left-HA-1.jpg]

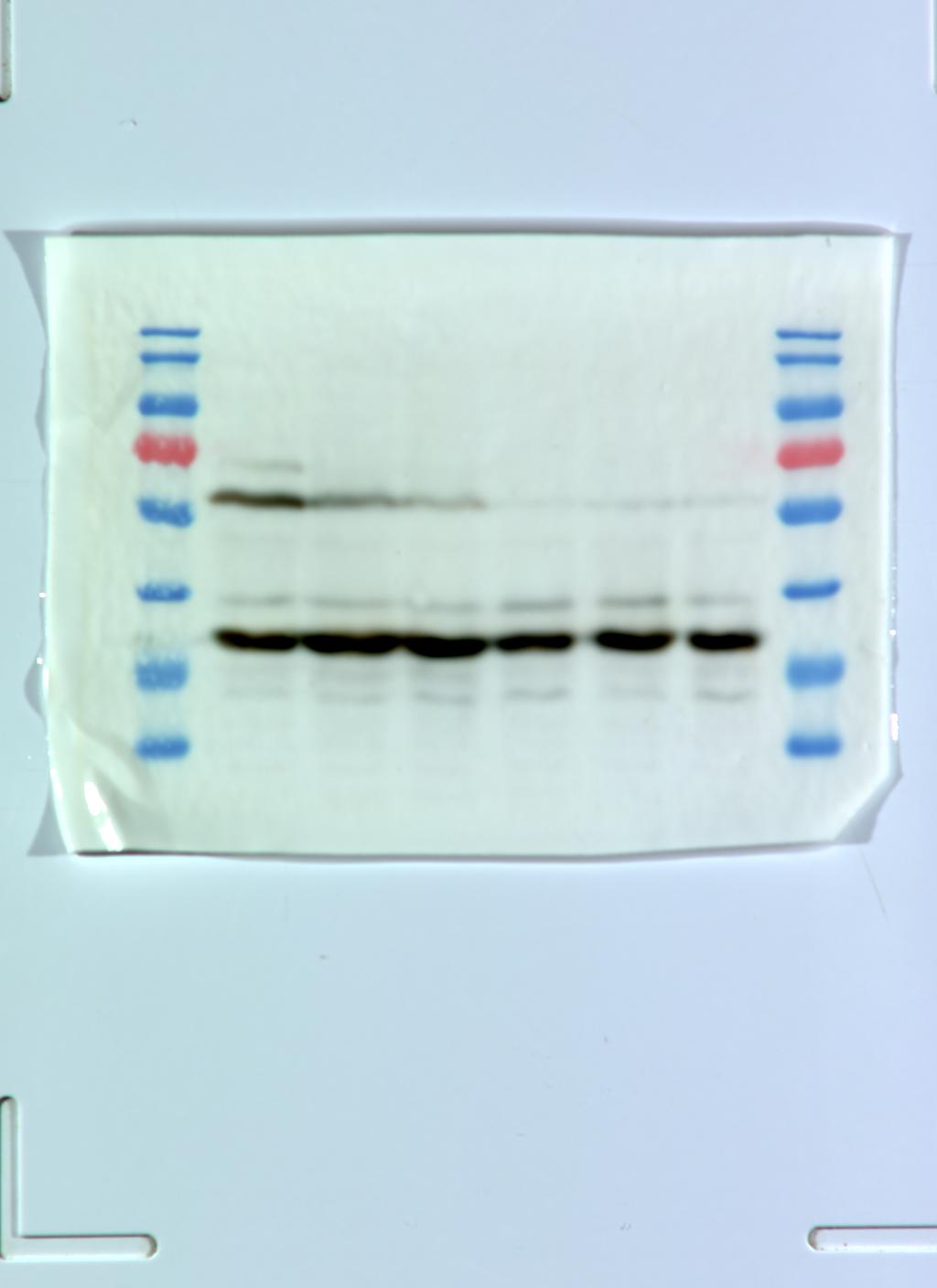

Supplement: Figure 6—source data 1. [file elife-80760-fig6-data1.zip › Figure 6-source data 1/Fig6D-right-GAPDH-2.jpg]

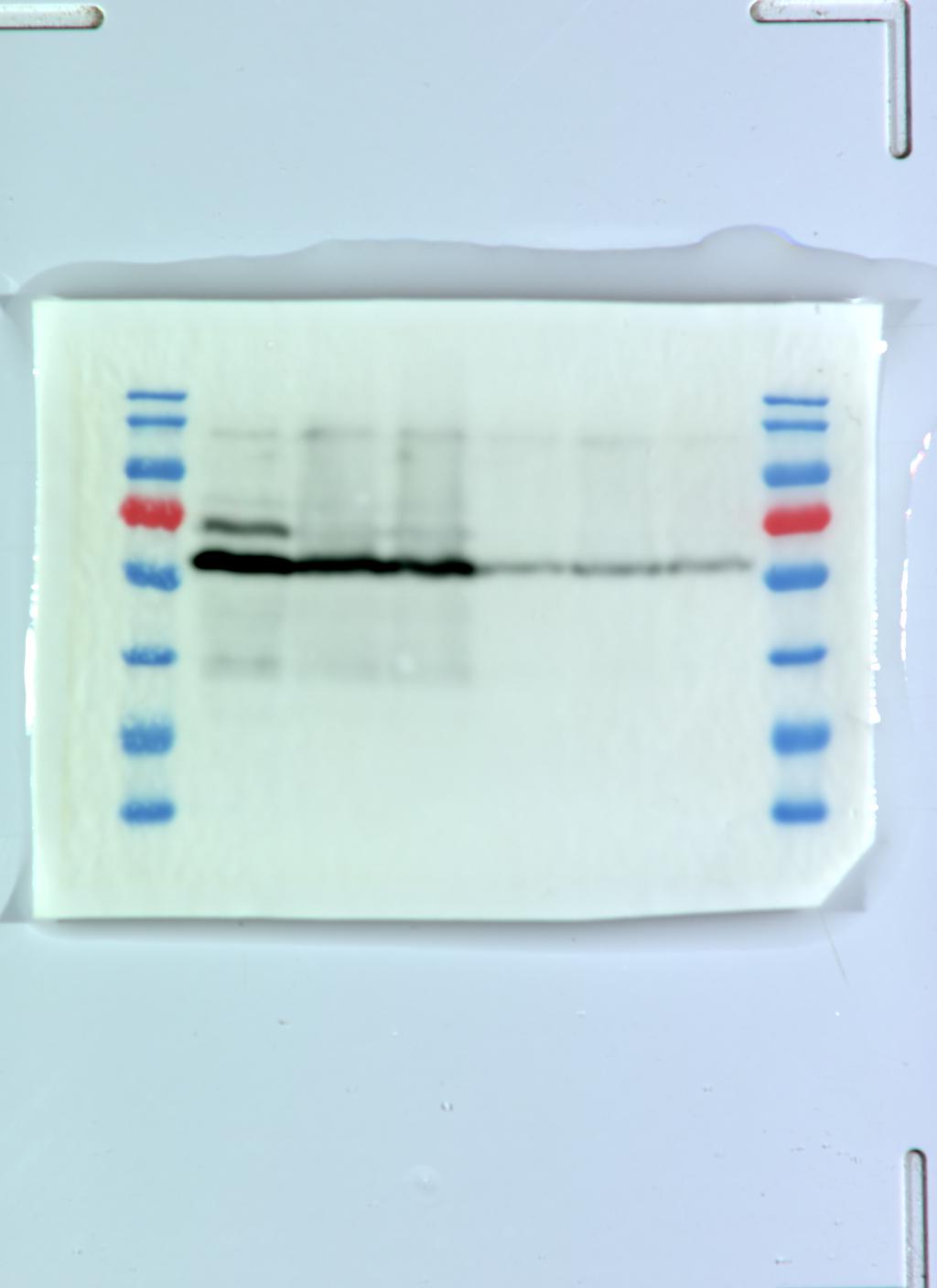

Supplement: Figure 6—source data 1. [file elife-80760-fig6-data1.zip › Figure 6-source data 1/Fig6D-right-HA-2.jpg]

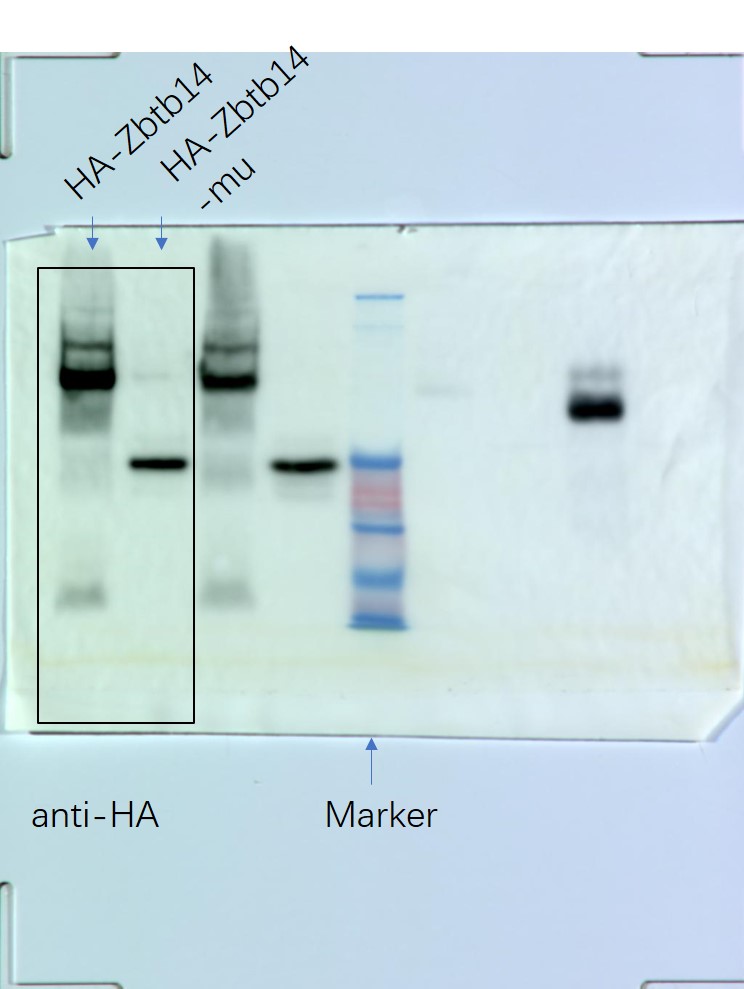

Supplement: Source data 1. [file elife-80760-data1.zip › Fig 1D.jpg]

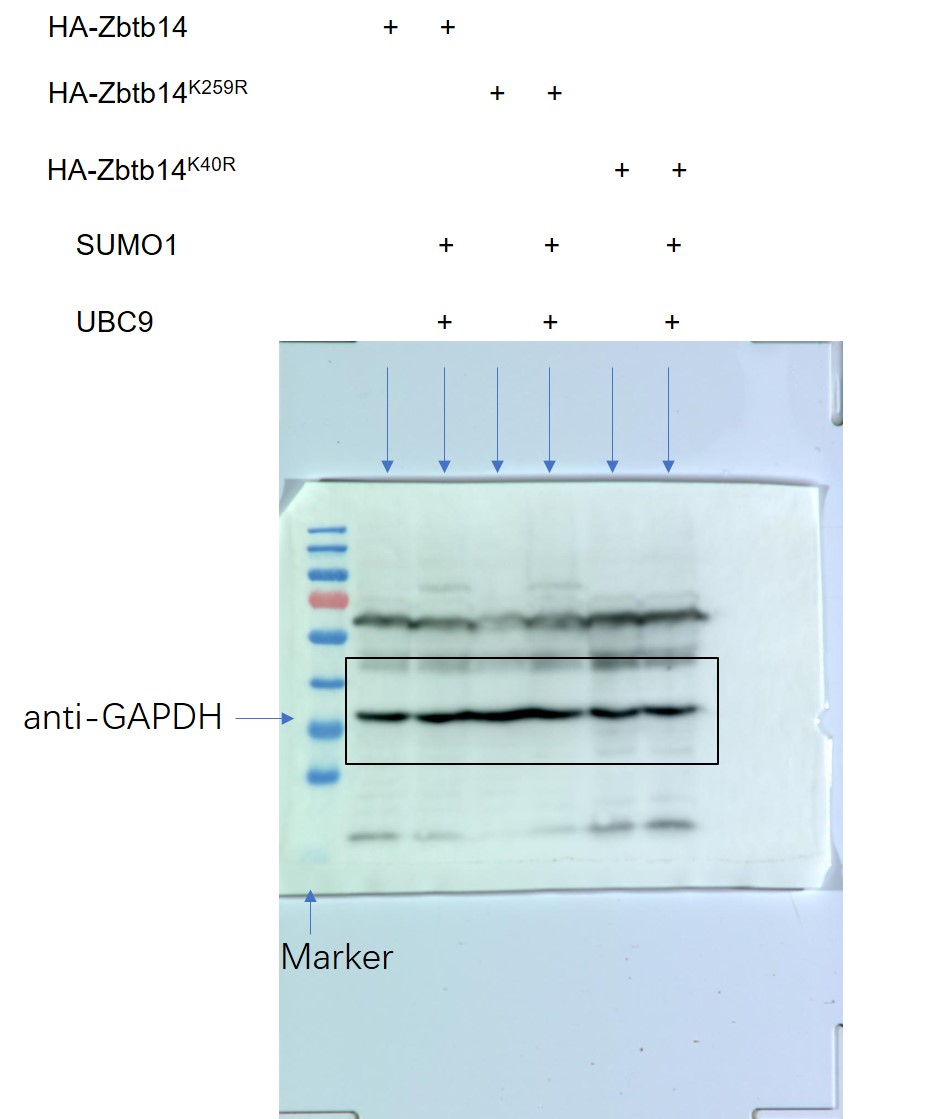

Supplement: Source data 1. [file elife-80760-data1.zip › Fig 5A-anti-GAPDH.jpg]

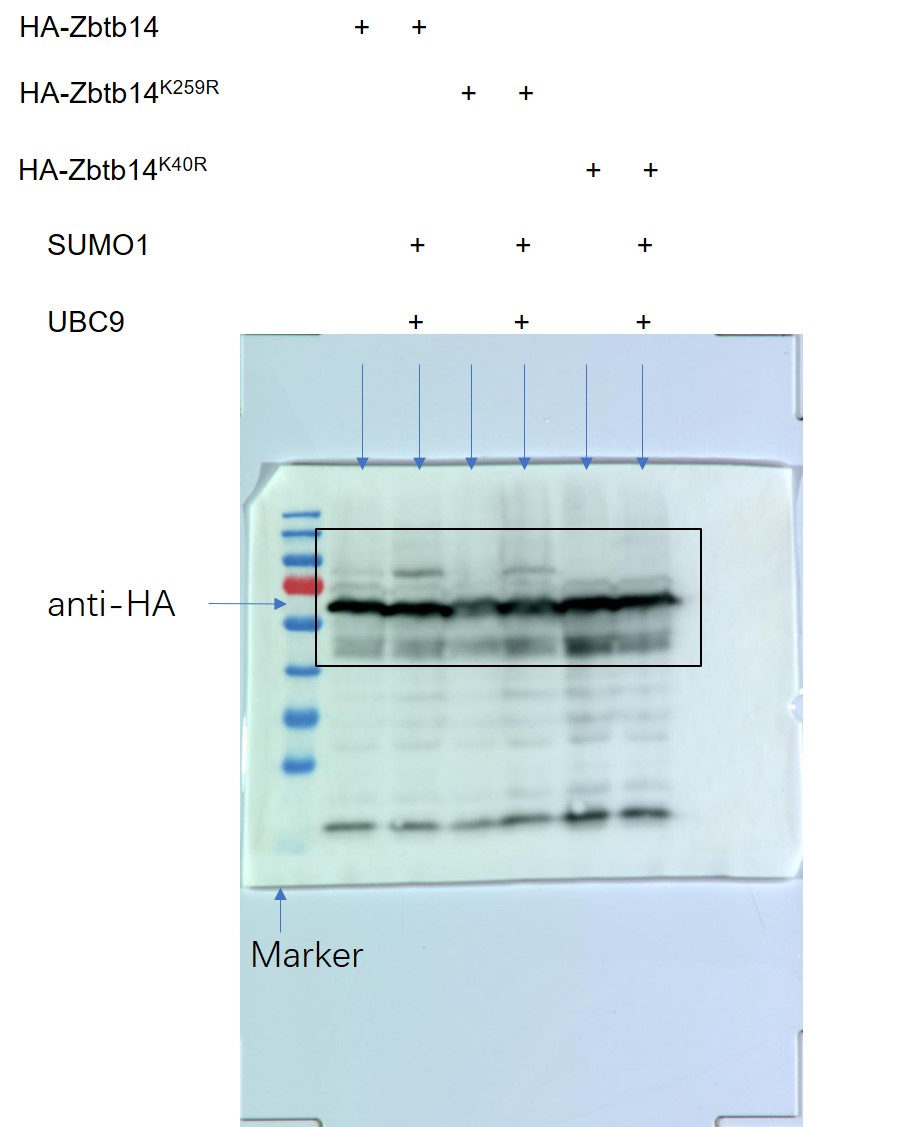

Supplement: Source data 1. [file elife-80760-data1.zip › Fig 5A-anti-HA.jpg]

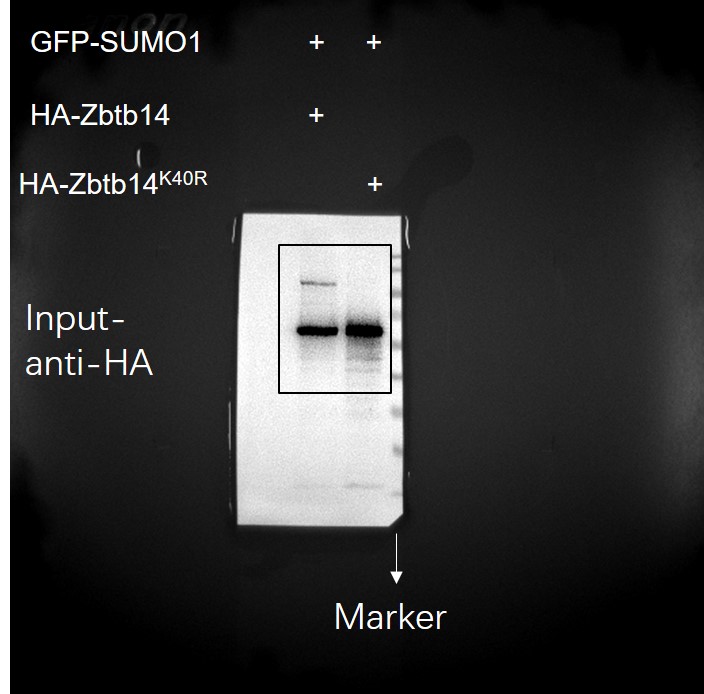

Supplement: Source data 1. [file elife-80760-data1.zip › Fig 5B-input-anti-HA.jpg]

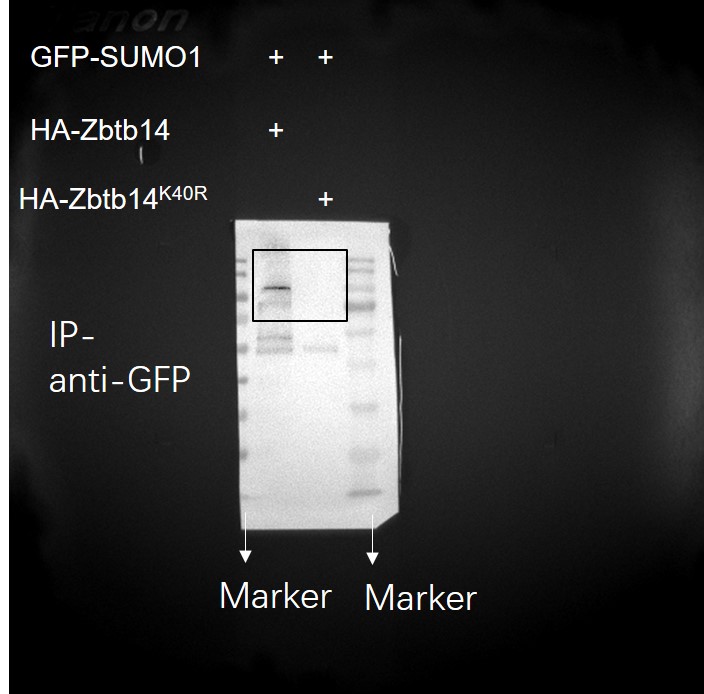

Supplement: Source data 1. [file elife-80760-data1.zip › Fig 5B-IP-anti-GFP.jpg]

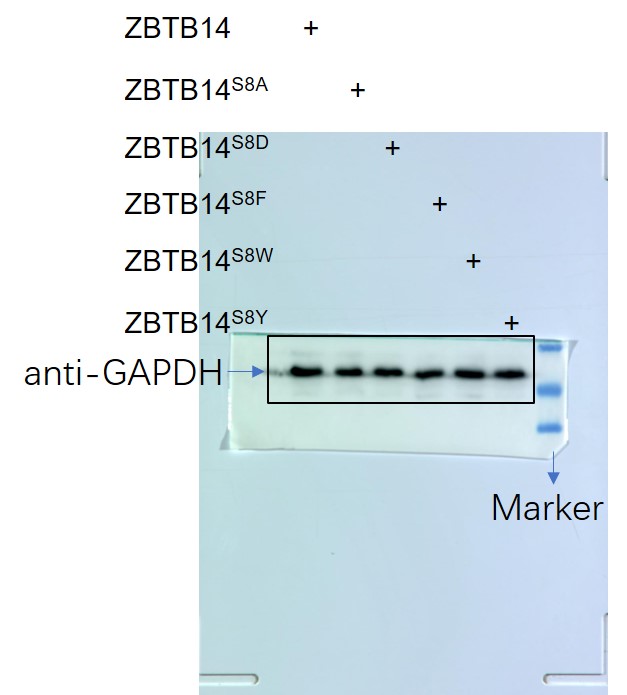

Supplement: Source data 1. [file elife-80760-data1.zip › Fig 6D-left anti-GAPDH.jpg]

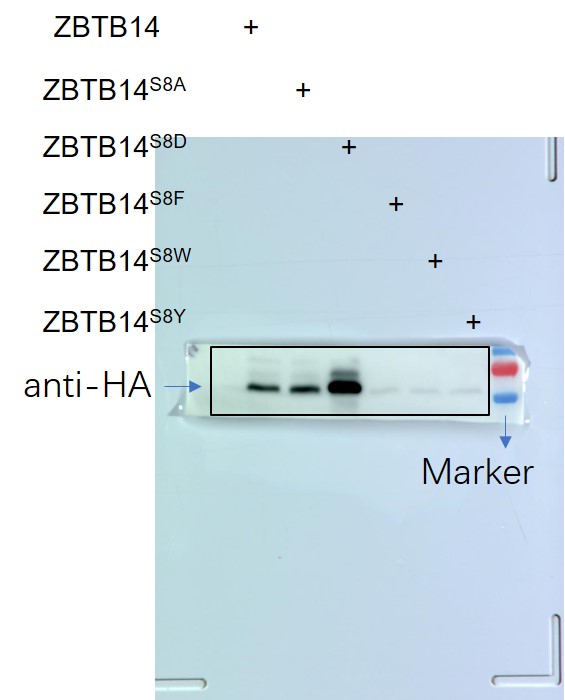

Supplement: Source data 1. [file elife-80760-data1.zip › Fig 6D-left anti-HA.jpg]

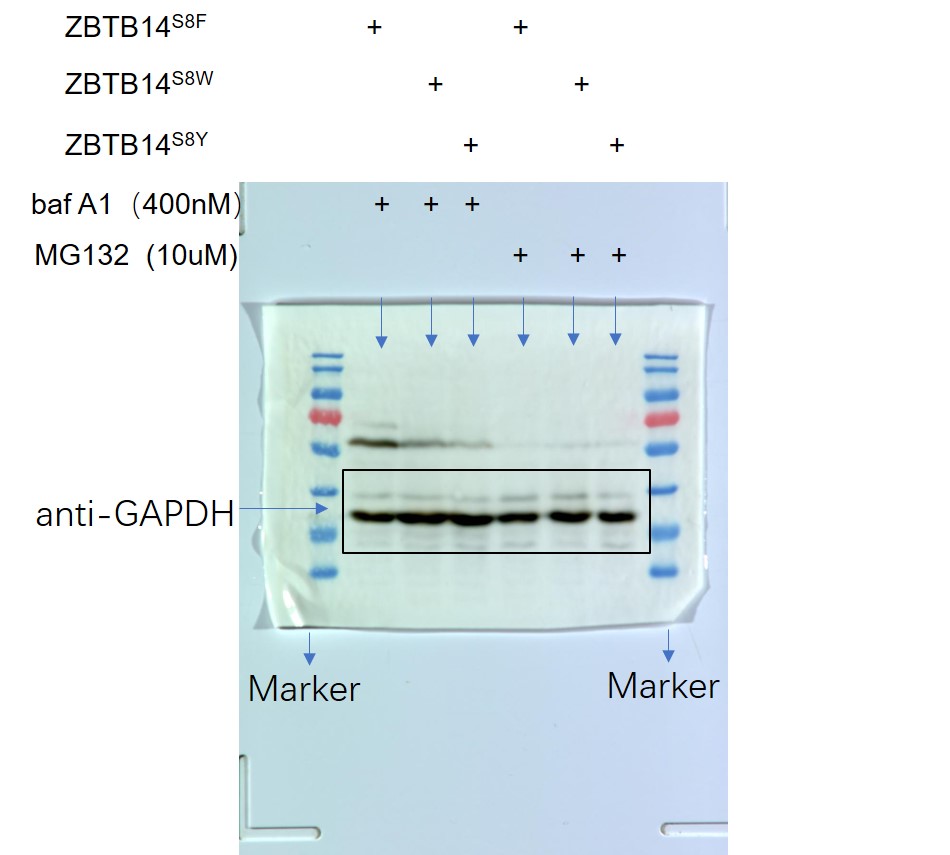

Supplement: Source data 1. [file elife-80760-data1.zip › Fig 6D-right-anti-GAPDH.jpg]

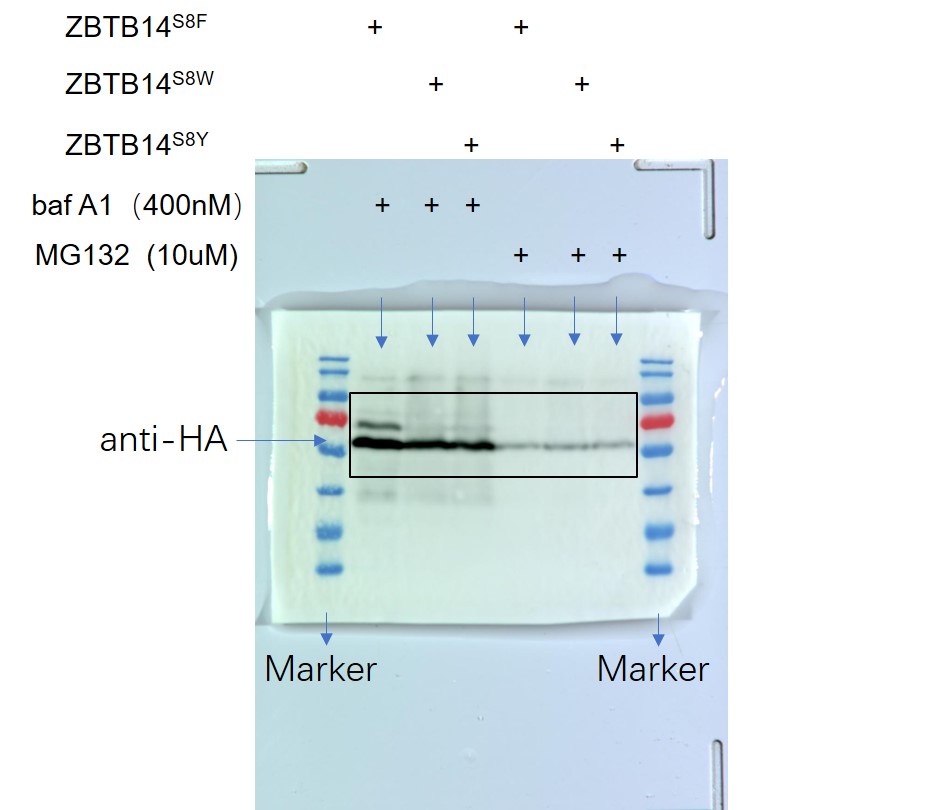

Supplement: Source data 1. [file elife-80760-data1.zip › Fig 6D-right-anti-HA.jpg]
